# Large Renal Calculi

**Published:** 1914-06

**Authors:** 


**Large Renal Calculi.** A. Grave of Moscow, quoted by Int. Jour. of Surg., reports a woman aged 49, whose kidney was removed on account of the destruction due to two calculi. The larger weighed nearly 12 ounces. This is considered the record case in Russian medical literature but the author cites Shield's case, 18 ounces and Dontu's, 34 ounces.

